# Evaluation of SARS-CoV-2 Antibody Response Between Paired Fingerprick (HemaPEN^®^) and Venepuncture Collected Samples in Children and Adults

**DOI:** 10.3390/antib14010013

**Published:** 2025-02-05

**Authors:** Nadia Mazarakis, Zheng Quan Toh, Jill Nguyen, Rachel A. Higgins, James Rudge, Belinda Whittle, Nicholas J. Woudberg, Justin Devine, Andrew Gooley, Florian Lapierre, Nigel W. Crawford, Shidan Tosif, Paul V. Licciardi

**Affiliations:** 1 Infection, Immunity and Global Health, Murdoch Children’s Research Institute, Melbourne, VIC 3052, Australia; nadia.mazarakis@mcri.edu.au (N.M.); zheng.quantoh@mcri.edu.au (Z.Q.T.);; 2 Department of Paediatrics, The University of Melbourne, Melbourne, VIC 3052, Australia; 3 Trajan Scientific and Medical, Melbourne, NSW 2153, Australia; 4 Synexa Life Sciences, Cape Town 7441, South Africa; 5 The Royal Children’s Hospital, Melbourne, VIC 3052, Australia

**Keywords:** hemaPEN, SARS-CoV-2, IgG, humoral immune responses, serosurveillance, dried blood spot (DBS)

## Abstract

Serological surveillance of severe acute respiratory syndrome coronavirus 2 (SARS-CoV-2) antibodies is important to monitor population COVID-19 immunity. Dried blood spots (DBS) are a valuable method for serosurveys, particularly in remote settings and in children. We compared the measurement of SARS-CoV-2 spike-specific IgG in paired blood samples collected using standard venepuncture (serum) and the hemaPEN^®^ microsampling DBS device from children and adults. A total of 83 participants (10 months to 65 years of age), comprising COVID-positive and -negative participants, were recruited. Paired serum and DBS samples were assayed for SARS-CoV-2 receptor-binding domain (RBD) and Spike (S1) antibodies using an established in-house ELISA. RBD and S1 IgG concentrations of paired hemaPEN DBS eluates and serum samples were compared using a non-parametric Wilcoxon matched-pairs signed ranked test. A Pearson’s correlation was used for RBD and S1 IgG concentrations and the level of agreement between the hemaPEN DBS eluates and serum samples was assessed by Bland–Altman analysis. A total of N = 41 adults (36 COVID-positive and 5 COVID-negative), and N = 42 children (37 COVID-positive, and 5 COVID-negative) have paired serum and DBS assayed. We found moderate to strong correlations between paired hemaPEN DBS eluates and serum SARS-CoV-2 IgG antibodies for RBD (r = 0.9472, *p* < 0.0001) and S1 proteins (r = 0.6892, *p* < 0.0001). Similar results were observed in both adult and paediatric populations. No significant differences in S1-specific IgG levels were observed in hemaPEN DBS samples stored for up to 35 weeks at room temperature. Eluted hemaPEN samples showed high specificity and sensitivity (100% and 89.89%, respectively) compared with serum. The use of the microsampling hemaPEN device for DBS sample collection is a feasible approach for assessing SARS-CoV-2 antibodies for serosurveillance studies, particularly in remote settings and in children.

## 1. Introduction

Serological surveillance of severe acute respiratory syndrome coronavirus 2 (SARS-CoV-2) can provide estimates of population exposure and immunity, an important component to help inform the public health response. The emergence of new SARS-CoV-2 variants that escape immunity from earlier variants and waning immunity justifies the need for surveillance across the life course, with Australia having high seroprevalence following the Omicron wave in late 2021 and early 2022 [1]. Continued serosurveillance is particularly important in countries with low vaccine coverage and in young infants <2 years, who may not have been exposed to the SARS-CoV-2 virus.

Serology testing typically involves serum/plasma samples collected using venepuncture. However, this method requires access to trained staff, blood processing equipment, −20 °C freezer infrastructure, and transportation logistics to laboratory facilities, all of which can be costly and logistically challenging, especially in lower- to middle-income countries (LMIC) and remote settings. Fingerprick tests on dried blood spot (DBS) cards are a logistically feasible and less invasive approach than venepuncture. The DBS approach has been used to measure serological response in a number of infectious diseases and vaccines including measles [2], mumps, and rubella [3,4]. Furthermore, we and several other studies have demonstrated the measurement of SARS-CoV-2 antibodies in DBS collected on Gutherie cards [5,6,7,8,9,10]. However, there are some limitations to DBS samples collected on Guthrie cards such as sample volume variability, requirements to wait for cards to dry and appropriate storage to prevent contamination risk.

The hemaPEN^®^ is a novel microsampling device for the collection of DBS samples that offers some advantages over conventional DBS. The hemaPEN device uses a capillary mechanism to collect four volumetrically fixed DBS samples in a self-contained and easily storable way, which reduces the risk of contamination.

This study aimed to evaluate the use of the hemaPEN microsampling device for SARS-CoV-2 antibodies against paired venepuncture serum samples in adult and paediatric populations following SARS-CoV-2 infection and/or COVID-19 vaccination, using an established in-house ELISA. A secondary aim was to determine whether a Synexa assay platform can detect Spike-specific IgG and neutralising antibodies using DBS from the hemaPEN microsampling device and how well the Synexa assay correlates with the in-house ELISA.

## 2. Materials and Methods

### 2.1. Study Cohort

Participants were recruited as part of a household cohort study if they presented with a SARS-CoV-2 positive test (nasal/throat swab PCR-positive) at the Royal Children’s Hospital, Australia [11]. We also recruited participants who were SARS-CoV-2 negative and/or COVID-19 vaccinated only from the household cohort and a Health Care Worker vaccine cohort [12]. In both studies, data on COVID-19 PCR testing and vaccination records were collected. All COVID-19 among participants in our study were mild and did not require hospitalisation. Written informed consent and assent were obtained from adults/parents and children, respectively. Participants provided a fingerprick hemaPEN DBS sample as well as a venepuncture blood sample. This study was approved by the Royal Children’s Hospital Melbourne Human Research Ethics Committee (HREC): HREC/63666 and HREC 74705/RCHM-2019. Approval Date: 5 July 2021.

### 2.2. Sample Collection and Storage

**Figure 1:**
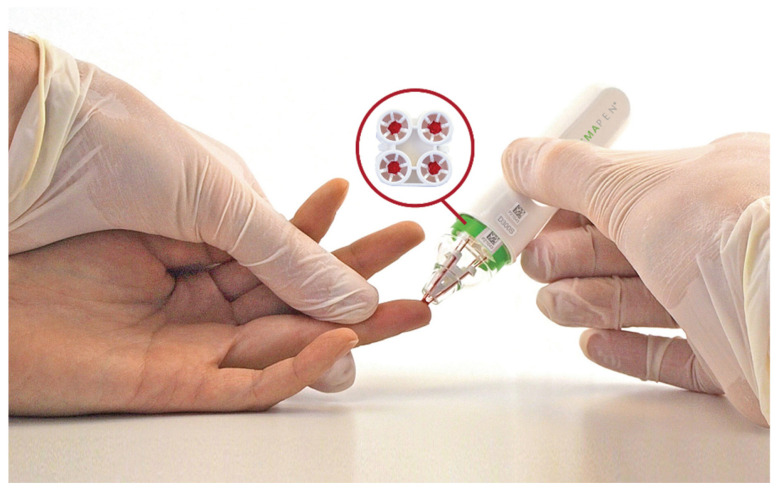
Illustration of the hemaPEN device in use. Following a fingerprick, blood is collected using the hemaPEN device onto four dried blood spot (DBS) discs. *Image acquired from Neoteryx by Trajan, accessed 11 November 2023 (https://www.neoteryx.com/media-download)*.

For the fingerprick sample collection, an alcohol swab was used to sanitise the finger, which was left to dry for 30 s before a lancet was used to create a drop of blood on the finger. The blood was collected with a hemaPEN device (Figure 1) that consists of four capillary tubes that transfer the blood onto four DBS discs, equivalent to 2.74 μL of blood/disc. HemaPEN collected samples were stored at room temperature at MCRI until analysis. A standard venepuncture procedure was used to collect blood samples. The blood samples were collected using SST gel tubes (Sarstedt, Nümbrecht Germany) and were processed within 2 h of collection to obtain serum by centrifugation at 2500× *g* for 10 min. The serum samples were stored at −80 °C.

### 2.3. Measurement of SARS-CoV-2 RBD- or S1-IgG Using In-House ELISA

Two DBS discs (each 3 mm disc = 3 μL) collected from the hemaPEN device were added to an Eppendorf tube and diluted in a total volume of 274 μL 10% skim milk-PBS-0.1% Tween (1:50 dilution). The Eppendorf tubes were placed on a plate shaker overnight (500 rpm at room temperature) to elute the antibodies [8]. An in-house SARS-CoV-2 ELISA was used for this study, which was previously compared with commercial assays, including DiaSorin LIAISON^®^ SARS-CoV-2 IgG Test and Wantai SARS-CoV-2 ELISA kit as previously reported [8,11]. Briefly, high-binding 96-well plates were coated with 2 μg/mL of receptor-binding domain (RBD) or S1 protein of the ancestral strain (Sino Biological, Beijing, China) diluted in phosphate-buffered saline (PBS). Plates were blocked with 10% skim milk-PBS-0.1%Tween, prior to the addition of paired serum (1:50) and hemaPEN DBS eluates (1:50) samples. A goat anti-human IgG (1:10,000) horseradish peroxidase–conjugated secondary antibody was used, and the plates were developed using 3.3′, 5.5′-tetramethylbenzidine substrate solution. Samples were first screened with the SARS-CoV-2 RBD antigen, and seropositive samples were confirmed and titrated with the S1 antigen. For RBD, results were reported as optical density (OD450 nm), with results greater than 0.5 OD units considered seropositive based on pre-pandemic controls [8]. These pre-pandemic serum samples were collected and stored at −80 °C from an ethically approved study (HREC 35253) and were used as a negative control to establish the seropositivity cut-off. For S1, results were converted to binding antibody units (BAU/mL) based on a World Health Organization SARS-CoV-2 pooled serum standard (National Institute of Biological Standards and Controls, Ridge, UK). The cut-off for seropositivity was 8.82 BAU/mL. The cut-offs values for RBD and S1 were based on our previously optimised methodology that demonstrated a strong correlation between paired DBS and serum samples [8].

### 2.4. Measurement of SARS-CoV-2 Antibodies Using Synexa Assay

A total of N = 150 DBS discs were sent to Synexa (Cape Town, South Africa) from N = 83 participants (up to two hemaPEN DBS discs per participant) for the measurement of SARS-CoV-2 antibodies. Analysis of samples at Synexa was undertaken 32 weeks after collection, and the samples were stored at Synexa at −20 °C prior to analysis. Control samples (N = 7 negative SARS-CoV-2 and N = 3 convalescent hemaPEN samples) were provided by Synexa in addition to the DBS samples.

Samples and controls were eluted according to Synexa’s DBS sample processing protocol, as detailed in Maritz et al., 2022 [6]. Briefly, the DBS samples were eluted into LowCross BufferR^®^ (Candor Bioscience, Allgäu, Germany) at 350 ± 50 RPM overnight at 2–8 °C and, following elution, stored at −70 °C before analysis. The Synexa assay measures IgG specific for the SARS-CoV-2 spike protein and neutralising antibodies (NAb) using a surrogate neutralising competitive assay. Assay wells were coated with the wildtype S1 spike protein. The IgG-specific assay used an anti-human IgG secondary antibody for detection. The neutralising assay detected antibodies with HRP-conjugated ACE-2. These assays are semi-quantitative, with an assay cut point (ACP) calculated based on the mean signal of the negative controls, as previously described [6]. Binding IgG antibodies were considered positive above the ACP cut-off, and for NAb, signals below the ACP cut-off were deemed positive.

### 2.5. Statistical Analysis

The RBD and S1 IgG concentrations of paired hemaPEN DBS eluates and serum samples were compared using a non-parametric Wilcoxon matched-pairs signed ranked test. The proportion of participants who were IgG seropositive to RBD and S1 (±95% CI) were described. For correlation analyses, a Pearson’s correlation was used for RBD and S1 IgG concentrations, as well as for the in-house ELISA and Synexa assay. The level of agreement between the two methods used for collecting samples (the hemaPEN DBS eluates and serum samples) was assessed by Bland–Altman analysis [13]. The sensitivity of serum and DBS sampling for the detection of RBD and S1-specific antibodies using the MCRI In-house ELISA assay was reported. Statistical analyses were performed using GraphPad Prism 9.1.1 (GraphPad Software Inc., San Diego, CA, USA), with a two-sided *p* < 0.05 considered statistically significant.

## 3. Results

### 3.1. Characteristics of Participants

A total of N = 83 participants (10 months to 65 years of age) were recruited (Table 1) between July and December 2021. Participants were defined as being positive for SARS-CoV-2 antibodies either by infection defined as RT-PCR-positive, a history of COVID-19 vaccination, or a combination of infection and vaccination. Additionally, participants who were defined as being negative for SARS-CoV-2 antibodies were confirmed with RT-PCR and grouped as uninfected/unvaccinated.

### 3.2. Comparison of SARS-CoV-2 RBD and S1 Antibody Responses Between HemaPEN DBS Eluates and Serum Samples

**Figure 2:**
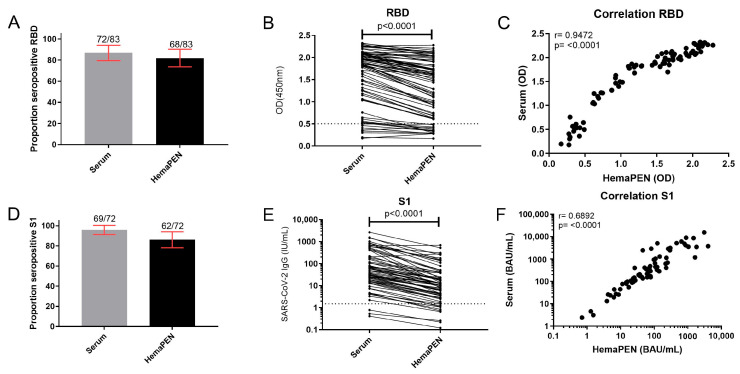
Comparison of paired hemaPEN DBS eluates and serum specimens for SARS-CoV-2 IgG specific for the RBD and S1 proteins of SARS-CoV-2. An in-house ELISA to measure IgG concentrations specific for the receptor-binding domain (RBD) of the SARS-CoV-2 spike protein was used to determine (**A**) the number of seropositive samples for RBD, (**B**) the concentration of RBD-specific IgG, and (**C**) a correlation between paired hemaPEN DBS eluates and serum specimens for RBD-specific IgG. Seropositive results for RBD-specific IgG were then assayed for IgG specific for the S1 antigen of the SARS-CoV-2 spike protein. (**D**) The proportion of IgG seropositive samples for S1, (**E**) the concentration of S1-specific IgG, and (**F**) corresponding correlations between paired specimens using a Pearson’s correlation test for S1-specific IgG. A non-parametric Wilcoxon matched-pairs signed ranked test was used for statistical analysis with a two-sided *p* < 0.05 considered statistically significant. The cut-off for seropositivity is represented by the dotted line. OD: optical density. BAU: binding antibody units. r: correlation coefficient.

Between paired serum and hemaPEN DBS eluates, the proportion of seropositive samples was not significantly different for RBD and S1 (Figure 2A and Figure 2D, respectively). The hemaPEN DBS eluates had significantly lower IgG concentrations specific for RBD and S1 (*p* < 0.0001) compared to serum (Figure 2B and Figure 2E, respectively). Moderate to strong correlations were observed for both SARS-CoV-2 RBD (r = 0.9472, *p* < 0.0001) and S1-specific IgG S1 (r = 0.6892, *p* < 0.0001) between paired hemaPEN eluted DBS and serum (Figure 2C and Figure 2F, respectively).

### 3.3. Comparison of SARS-CoV-2 Antibody Responses Between hemaPEN DBS Eluates and Serum Samples Within Children and Adults

**Figure 3:**
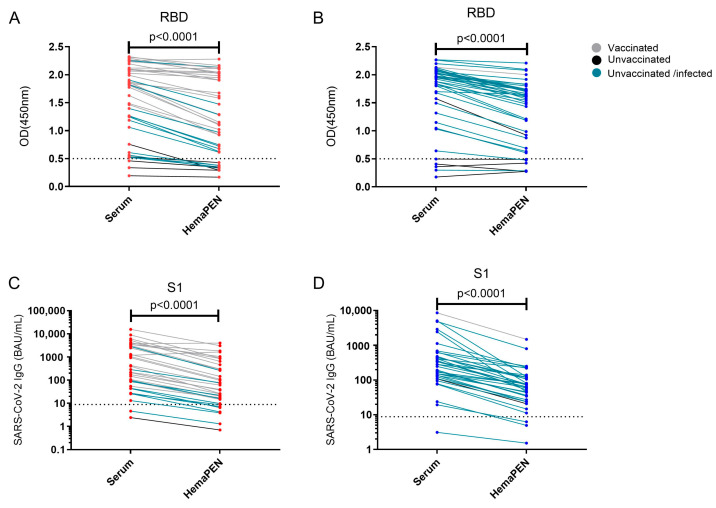
Comparison of paired hemaPEN DBS eluates and serum specimens for SARS-CoV-2 IgG specific for the RBD and S1 protein in adults and children. An in-house ELISA was used to measure IgG concentrations specific for the receptor-binding domain (RBD) and S1 of the SARS-CoV-2 spike protein between paired hemaPEN DBS eluates and serum specimens in adults (**A**,**C**), respectively and children (**B**,**D**), respectively. Individuals who have received at least one dose of the COVID-19 vaccine are shown as grey lines, individuals who did not receive the COVID-19 vaccine are shown as black lines and individuals who were unvaccinated/infected with SARS-CoV-2 are shown as blue lines. A non-parametric Wilcoxon matched-pairs signed ranked test was used for statistical analysis with a two-sided *p* < 0.05 considered statistically significant. The cut-off for seropositivity is represented by the dotted line. OD: optical density. BAU: binding antibody units.

To assess whether there is a difference in the measurement of SARS-CoV-2 antibody response using the two sampling methods in paediatric and adult samples, we stratified our analysis into children and adult populations. Similar results were observed for both adults (Figure 3A,C) and children (Figure 3B,D), with a significant decrease (*p* < 0.001) in SARS-CoV-2 RBD and S1 IgG response for hemaPEN DBS eluates compared to paired serum samples. COVID-19-vaccinated individuals had higher concentrations of RBD and S1-specific IgG compared to the unvaccinated group in both adults (Figure 3A,C) and children (Figure 3B,D). Similar results were also observed in different immune states: vaccinated only, infected only, and vaccination and infected cohorts (Supplementary Figure S1).

### 3.4. Effect of Storage Time and Temperature

**Figure 4:**
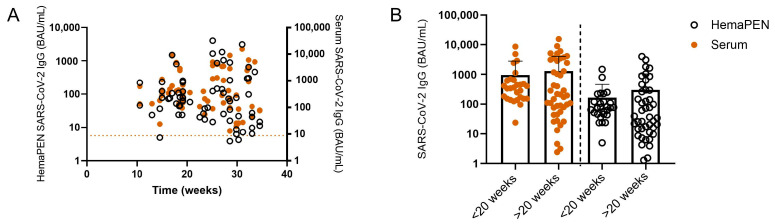
Effect of storage time on IgG specific for S1 protein for paired hemaPEN DBS eluates and serum samples. (**A**) hemaPEN DBS storage time and SARS-CoV-2 IgG concentrations in hemaPEN DBS eluates (open circle) or serum (orange circle). (**B**) SARS-CoV-2 IgG concentrations in hemaPEN DBS eluates (open circle) or serum (orange circle) stratified by storage time less than or greater than 20 weeks. Results are displayed as mean with standard deviation. A non-parametric Wilcoxon matched-pairs signed ranked test was used for statistical analysis with a two-sided *p* < 0.05 considered statistically significant. The cut-off for seropositivity is represented by the dotted line. BAU: Binding antibody units.

To evaluate the stability of the DBS from the hemaPEN microsampling device, we compared IgG specific for S1 SARS-CoV-2 antigen between paired hemaPEN DBS eluates and serum samples over time (Figure 4). Analysis of samples at MCRI occurred between 10 and 35 weeks (median 27 weeks) after sample collection (Figure 4A). No significant differences in S1-specific IgG levels were observed when samples were assayed less than 20 weeks or more than 20 weeks after collection (Figure 4B).

### 3.5. Sampling Agreement Between Serum and HemaPEN DBS Samples

**Figure 5:**
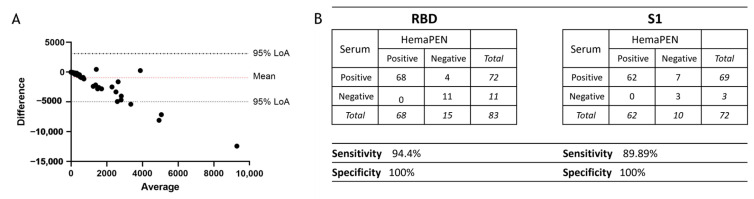
(**A**) Bland–Altman plot of IgG concentrations specific for S1 antigen of SARS-CoV-2 and (**B**) sensitivity and specificity calculations amongst paired specimens for RBD and S1 specific IgG concentrations. The grey dotted lines represent the 95% limits of agreement.

A Bland–Altman analysis was used to compare the two blood collection methods where the ‘true’ value in the sample is unknown. Both sampling methods displayed a high level of agreement, with 95.18% of samples within the 95% limits (Figure 5A). The sensitivity and specificity for the hemaPEN DBS eluates to measure SARS-CoV-2 RBD IgG were 94.40% and 100%, respectively, when compared to serum specimens. For the detection of S1-specific IgG, the hemaPEN DBS eluates had a sensitivity specificity of 89.89% and 100%, respectively.

### 3.6. Synexa Assay Analysis on HemaPEN DBS Specimens

Binding antibodies for the S1 SARS-CoV-2 protein were found to have a good correlation amongst the hemaPEN DBS eluates assayed on the Synexa assay versus paired hemaPEN DBS assayed at MCRI (Supplementary Figure S2A, r = 0.6059, *p* < 0.0001). Seropositivity between binding S1 IgG assay platforms was comparable, with no significant differences (Supplementary Figure S2B). The Synexa also measured NAb based on competition, with high OD representing a low NAb response. A negative correlation was observed with NAb and binding IgG antibodies with hemaPEN DBS eluates using the Synexa assay (Supplementary Figure S2C, r = −0.7842, *p* < 0.0001). The proportion of positive NAb in the hemaPEN DBS eluates was higher in children (23/41; 56.1%) compared to adults (12/39; 30.8%) (Supplementary Figure S2D).

## 4. Discussion

Our results demonstrate that the DBS eluates from the novel sampling device, hemaPEN, had homogeneity with paired venepuncture serum samples and correlated well for detecting SARS-CoV-2 RBD and S1 IgG. Despite the lower antibody concentrations observed in hemaPEN eluates when compared to serum, we found high sensitivity and specificity for the detection of SARS-CoV-2 spike antibodies in both paediatric and adult cohorts, providing confidence for the use of this microsampling device.

Our data indicates that hemaPEN sampling is best suited for individuals or populations with high levels of SARS-CoV-2 immunity and may be used for serosurveillance to help inform public health responses, since the proportion of seropositive by the two sampling methods were similar. However, compared with serum samples, we found hemaPEN DBS eluates have lower sensitivity for samples with lower/borderline IgG concentrations, which might result in false negative results. Nevertheless, the lower IgG concentrations indicate low SARS-CoV-2 population immunity overall, highlighting the need for public health measures. The lower IgG concentrations in hemaPEN DBS were unexpected as we previously showed a strong correlation between SARS-CoV-2 RBD and S1 antibody levels between DBS collected via Guthrie card (Whatman 903 filter paper) and paired serum samples [8]. Other studies have also reported strong correlations between SARS-CoV-2 neutralising antibodies and binding antibodies between DBS and paired serum samples [5,14,15]. These results are different from our negative correlation observed between the hemaPEN DBS eluates and the serum samples with the Synexa assay. This difference is likely due to the different assay platforms, whereby the Synexa assay is a competition assay and/or due to antibody saturation in the samples. However, comparisons between studies are difficult as different elution buffers and elution volumes, as well as sample dilutions and types of antibody assays, were used. For hemaPEN DBS, further optimisation on the assay cut-offs and DBS elution volume may be needed to improve sensitivity and specificity. In addition, it would also be interesting to examine whether DBS samples can be used to characterise antibody affinity and antibody-effector function and to explore the potential use of detection of other SARS-CoV-2 specific antibodies to newer and/or current variants.

Serosurveys remains an important component of the public health response against COVID-19 [16,17]. Serosurveys monitor population immunity to SARS-CoV-2 and inform public health measures, including the administration of booster doses. The advantage of using DBS samples for serosurveillance is evident because it provides a logistically more feasible approach by reducing the need for cold-chain transportation, highly trained personnel and facilities. These factors are particularly relevant for remote settings in high-income countries and/or LMICs or at-home settings for self-collection where there are logistical barriers. The hemaPEN device offers an easier way to collect and store blood samples than a traditional Guthrie card for immunological assessment. This is achieved through the capillary mechanism that enables the collection of volumetrically fixed samples and allows the samples to dry within the device, reducing the risk of contamination [18,19,20]. Furthermore, we demonstrated acceptable stability of the hemaPEN DBS eluates to be stored for up to a minimum of 35 weeks at room temperature, a major advantage for remote settings. In terms of compatibility with existing laboratory infrastructure for processing DBS specimens, no specialised equipment is needed for the hemaPEN device.

In addition to assessing sample sensitivity, evaluating the user experience and patient feasibility is important. Personal communication about the use of the hemaPEN device from both adults and parents of paediatric participants was well received due to its efficiency and minimal blood collection. It was the preferred method of choice compared to venepuncture, particularly for children. Good clinical practice was instrumental in ensuring a successful and comfortable patient experience. Furthermore, the user-friendly nature of the hemaPEN microsampling device makes it a feasible option for self-collection applications when needed.

This study has a few limitations. The sample size was small, yet the advantage of this study was that it included both a paediatric and adult cohort. Furthermore, sample collection was undertaken opportunistically at either one or 12 months after the initial household member presented positive for COVID-19. This meant that samples were collected for antibody responses relative to the time of COVID-19 infection and not to the time of COVID-19 vaccination. Nonetheless, the results observed in this study are consistent and demonstrate the validity of the hemaPEN device for the successful detection of SARS-CoV-2 antibodies.

## 5. Conclusions

In conclusion, we showed that the hemaPEN device may be an alternative to venepuncture for blood collection to measure SARS-CoV-2 immunity. This device will be relevant for serosurveillance studies and populations (i.e., children) where a less invasive sampling is preferred. While we used this device to measure SARS-CoV-2 immunity, the device can also be used to monitor immunity to other infectious diseases, such as mumps and rubella, where DBS approaches have been widely used.

## Figures and Tables

**Table 1:** Participant’s characteristics.

	Adult (N = 41)	Children (N = 42)
Age in years, median (IQR)	38 (26–65)	5 (0–18)
Infected, N (%)	13 (31.7%)	36 (85.7%)
Time since last documented infection (weeks), median (IQR)	52 (51.9–52.1)	5.1 (4.6–7.3)
Vaccinated, N (%)	16 (39%)	1 (2.4%)
Time since last vaccine dose (weeks), median (IQR)	6.3 (4.0–13.5)	8.1
Infected and vaccinated, N (%)	7 (17.1%)	-
Negative, N (%)	5 (12.2%)	5 (11.9%)

## Data Availability

The original contributions presented in the study are included in the article/Supplementary Materials. Further inquiries can be directed to the corresponding author.

## Supplementary Materials

The following supporting information can be downloaded at: https://www.mdpi.com/article/10.3390/antib14010013/s1, Supplementary Figure S1. Comparison of paired hemaPEN DBS eluates and serum specimens for SARS-CoV-2 IgG specific for the RBD and S1 protein in different immune state subgroups. An in-house ELISA was used to measure IgG concentrations specific for the receptor-binding domain (RBD) and S1 of the SARS-CoV-2 spike protein between paired hemaPEN DBS eluates and serum specimens in (A) vaccinated only cohort, (B) infected only cohort, and (C) vaccinated and infected cohort. A nonparametric Wilcoxon matched-pairs signed ranked test was used for statistical analysis with a two-sided *p* < 0.05 considered statisti-cally significant. The cut-off for seropositivity is represented by the dotted line. OD: optical density. BAU: Binding antibody units. Supplementary Figure S2. Synexa analysis of hemaPEN DBS eluates for binding IgG specific for S1 antigen of SARS-CoV-2 (A and B) and neutralising antibodies (NAb) (C and D). A Pearson’s correlation test was used for correlation analysis between hemaPEN DBS eluates IgG results obtained from Synexa and MCRI (A), and between the hemaPEN DBS neutralising antibody and IgG results obtained from Synexa (C). OD: optical density. BAU: Binding antibody units. r: correlation coefficient.
