# Supplementary material for: Evaluation of SARS-CoV-2 Antibody Response Between Paired Fingerprick (HemaPEN®) and Venepuncture Collected Samples in Children and Adults
Source: Antibodies (Basel). 2025 Feb 5;14(1):13. doi: 10.3390/antib14010013 (PMC11843976; doi:10.3390/antib14010013)
Supplement: Supplementary file 1 [file antibodies-14-00013-s001.zip › antibodies-3265221-supplementary.pdf]

Supplementary Materials

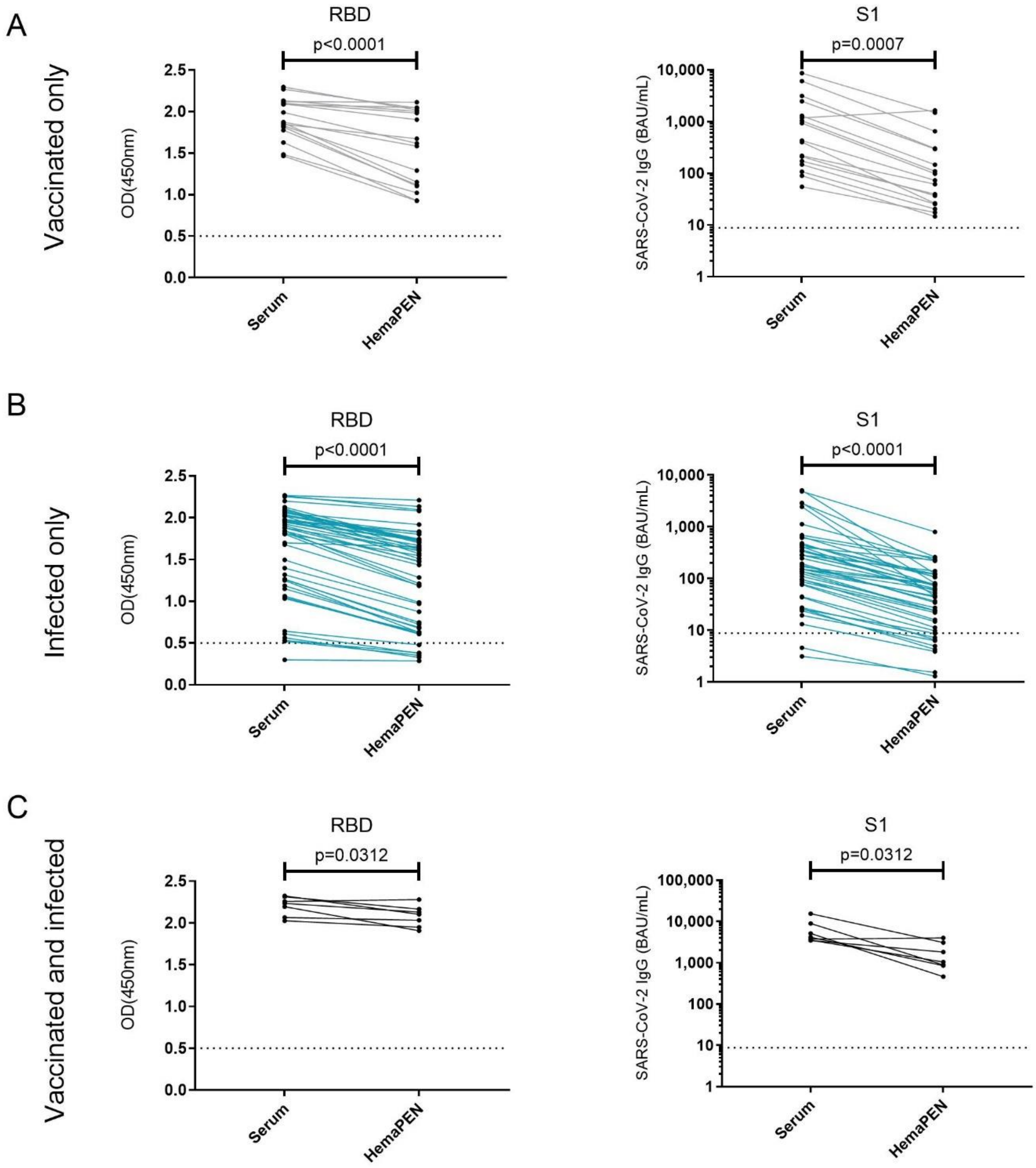

Supplementary figure 1. Comparison of paired hemaPEN DBS eluates and serum specimens for SARS-CoV-2 IgG specific for the RBD and S1 protein in different immune state subgroups. An in-house ELISA was used to measure IgG concentrations specific for the receptor-binding domain (RBD) and S1 of the SARS-CoV-2 spike protein between paired hemaPEN DBS eluates and serum specimens in (A) vaccinated only cohort, (B) infected only cohort, and (C) vaccinated and infected cohort. A nonparametric Wilcoxon matched-pairs signed ranked test was used for statistical analysis with a

two-sided P < .05 considered statisti-cally significant. The cut-off for seropositivity is represented by the dotted line. OD: optical density. BAU: Binding antibody units.

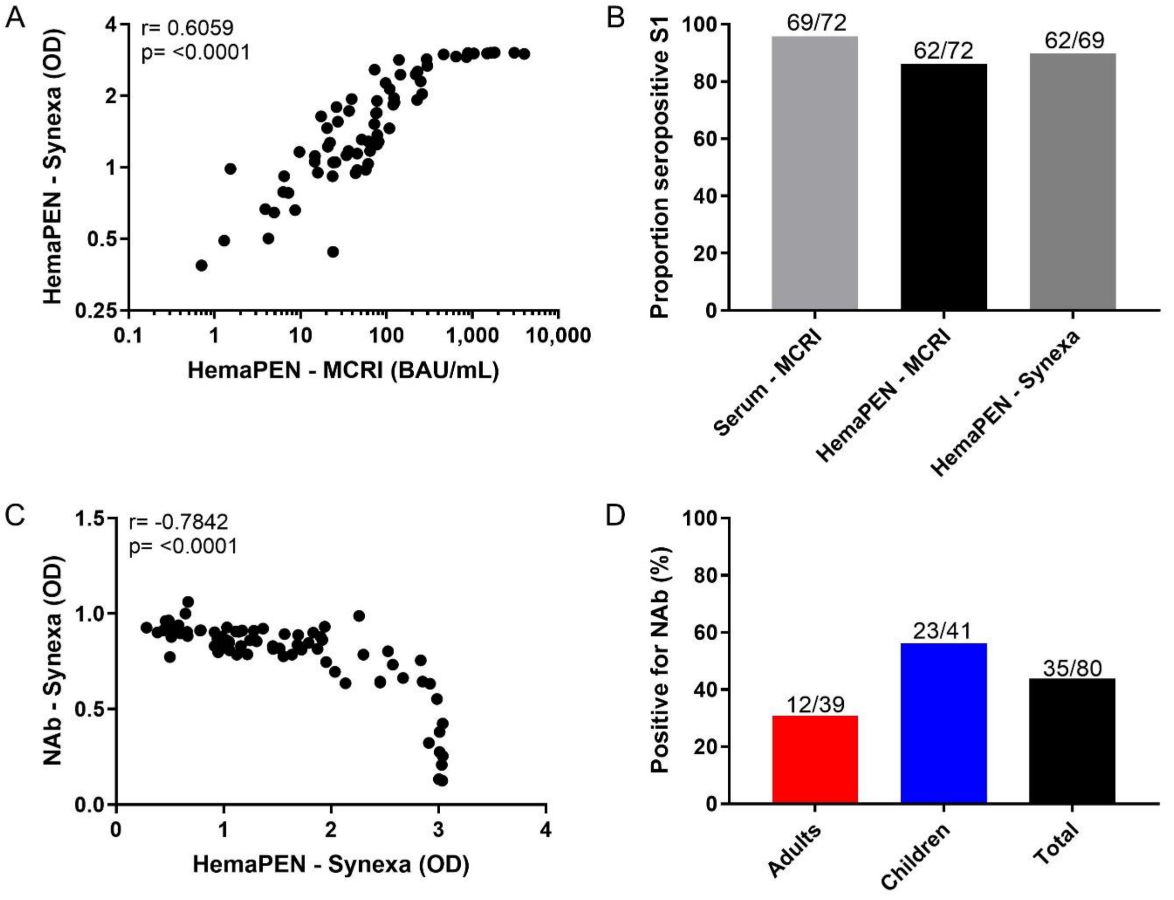

Supplementary figure 2. Synexa analysis of hemaPEN DBS eluates for binding IgG specific for S1 antigen of SARS-CoV-2 (A and B) and neutralising antibodies (NAb) (C and D). A Pearson's correlation test was used for correlation analysis between hemaPEN DBS eluates IgG results obtained from Synexa and MCRI (A), and between the hemaPEN DBS neutralising antibody and IgG results obtained from Synexa (C). OD: optical density. BAU: Binding antibody units. r: correlation coefficient.
